# Evolving CO_2_ Rather Than SST Leads to a Factor of Ten Decrease in GCM Convergence Time

**DOI:** 10.1029/2021MS002505

**Published:** 2021-10-30

**Authors:** Yixiao Zhang, Jonah Bloch‐Johnson, David M. Romps, Dorian S. Abbot

**Affiliations:** ^1^ Department of the Atmospheric and Oceanic Sciences Peking University Beijing China; ^2^ DNCAS‐Climate University of Reading Reading UK; ^3^ Department of Earth and Planetary Science University of California Berkeley CA USA; ^4^ Climate and Ecosystem Sciences Division Lawrence Berkeley National Laboratory Berkeley CA USA; ^5^ Department of the Geophysical Sciences University of Chicago Chicago IL USA

**Keywords:** planetary atmospheres, climate dynamics, global climate models

## Abstract

The high computational cost of Global Climate Models (GCMs) is a problem that limits their use in many areas. Recently an inverse climate modeling (InvCM) method, which fixes the global mean sea surface temperature (SST) and evolves the ${CO}_{2}$ mixing ratio to equilibrate climate, has been implemented in a cloud‐resolving model. In this article, we apply InvCM to ExoCAM GCM aquaplanet simulations, allowing the SST pattern to evolve while maintaining a fixed global‐mean SST. We find that InvCM produces the same climate as normal slab‐ocean simulations but converges an order of magnitude faster. We then use InvCM to calculate the equilibrium ${CO}_{2}$ for SSTs ranging from 290 to 340 K at 1 K intervals and reproduce the large increase in climate sensitivity at an SST of about 315 K at much higher temperature resolution. The speedup provided by InvCM could be used to equilibrate GCMs at higher spatial resolution or to perform broader parameter space exploration in order to gain new insight into the climate system. Additionally, InvCM could be used to find unstable and hidden climate states, and to find climate states close to bifurcations such as the runaway greenhouse transition.

## Key Points

- We converge a GCM by varying the CO_2_ while keeping the global‐mean surface temperature fixed (the inverse climate modeling method)
- Inverse climate modeling converges about 10 times faster than normal slab‐ocean simulations
- The SST gradient response timescale is the bottleneck on the convergence rate of inverse climate modeling

## 1 Introduction

The response of the climate to a change in ${CO}_{2}$ mixing ratio is a key question in the field of climate research. The equilibrium climate sensitivity (ECS) is defined as the increase in global‐mean surface temperature per doubling of ${CO}_{2}$ (IPCC, 1990). Several recent studies have demonstrated a robust increase in ECS in climates warmer than modern Earth due to state‐dependent feedback and forcing by ${CO}_{2}$ (Bloch‐Johnson et al., 2015; Bloch‐Johnson, Rugenstein, Stolpe, et al., 2020; Caballero & Huber, 2013; Colman & McAvaney, 2009; Hansen et al., 2005; Jonko et al., 2013; Leconte et al., 2013; Meraner et al., 2013; Popp et al., 2016; Romps, 2020; Russell et al., 2013; Seeley & Jeevanjee, 2021; Wolf & Toon, 2015; Wolf et al., 2018). When they investigate high enough temperatures, these studies find that the ECS reaches a maximum for global‐mean surface temperatures in the range of ∼310–320 K. We will refer to this as the “ECS bump.”

Detailed investigation of the ECS bump is limited by the computational expense of Global Climate Model (GCM) simulations. Inverse Climate Modeling (InvCM), which finds a set of parameters that can produce a given climate state rather than finding the equilibrium state produced by a given set of parameters, can significantly reduce computational cost. For example, Kasting (1988) assumed that the thermal structure of the atmosphere depends only on the surface temperature, so that the solar constant that achieves top‐of‐atmosphere (TOA) energy balance (known as the effective solar constant) can be diagnosed after calculating the planetary albedo and outgoing longwave radiation (OLR) for a given surface temperature. More recently, Romps (2020) introduced a method in which the ${CO}_{2}$ mixing ratio is prognostically evolved to balance the net TOA energy flux in a cloud‐resolving model with fixed sea surface temperature (SST) on a small domain. This method reduces the simulation time needed to reach convergence by at least an order of magnitude. In a cloud‐resolving model that runs on a small domain, the SST can be assumed to be uniform in space, which simplifies the implementation of this method. Romps (2020) speculated that this method could be applied to GCMs by fixing the global‐mean SST, but allowing the local SST to evolve in response to the local surface energy imbalance while the ${CO}_{2}$ is evolving in response to the global‐mean TOA energy imbalance.

The main purpose of this article is to implement InvCM in ExoCAM (Wolf & Toon, 2015; Wolf et al., 2018), a GCM specifically designed to be stable and accurate at high global‐mean surface temperatures and ${CO}_{2}$ levels. InvCM will allow us to investigate the ECS bump at high‐temperature resolution in SST, and we will also speculate about some future uses of InvCM in GCMs. In Section 2, we describe our implementation of InvCM in ExoCAM. In Section 3, we demonstrate the speed and accuracy of InvCM, show our ECS bump results, and demonstrate the possibility of finding unstable climate states. We discuss the possibility of equilibrating the model for a given SST pattern using InvCM, the linkage to conventional forward climate modeling, and the limitations of InvCM in Section 4. We give our conclusions in Section 5.

## 2 Methods

### 2.1 Inverse Climate Modeling

InvCM involves two steps. First, we fix one variable of the climate system, in this article the global‐mean SST. To do this, we need to apply a virtual flux. For example, we subtract the global‐mean surface energy flux from the local surface energy flux to fix the global‐mean SST. Second, we evolve one parameter of the model, such as the solar constant or ${CO}_{2}$ concentration, to adjust the virtual flux to zero so that the model equilibrates. Stated a different way, we evolve ${CO}_{2}$ in order to achieve global‐mean TOA energy balance while fixing the global‐mean SST and allowing the local SST to evolve in order to achieve local surface energy balance. In our slab‐ocean aquaplanet simulations, we evolve the local SST of the mixed‐layer ocean ($T_{s}$) with the following equation:

(1) $$\rho_{w}c_{w}H\frac{\partial T_{s}}{\partial t}=F_{s}-\bar{F_{s}}-\nabla \cdot \vec{q}$$

where $\rho_{w}$ and $c_{w}$ are the density and heat capacity of sea water; H is the mixed layer depth of the slab ocean; $F_{s}$ is the surface net downwelling energy flux; $\bar{F_{s}}$ is the global mean of $F_{s}$; and $\nabla \cdot \vec{q}$ is the ocean heat transport divergence. Note that $\nabla \cdot \vec{q}=0$ in all simulations in this article, but we include $\nabla \cdot \vec{q}$ in Equation 1 because it could be prescribed in future work.

The ${CO}_{2}$ mixing ratio, G, evolves with time as:

(2) $$\frac{d\log_{2}G}{dt}=-\frac{\bar{ASR}-\bar{OLR}}{\tau \cdot A_{0}}$$

where $A_{0}=3.7$ W $m^{-2}$. Here, τ is the ${CO}_{2}$ relaxation timescale, $\bar{ASR}$ and $\bar{OLR}$ represent the global‐mean absorbed shortwave radiation (ASR) and OLR. Note that G is spatially uniform throughout the atmosphere.

The convergence rate of InvCM is largely determined by the ocean mixed layer depth, H, and the ${CO}_{2}$ timescale, τ. H and τ are proportional to the timescale for local SST adjustment and the convergence of the TOA energy imbalance, respectively. Later, we will discuss how the choice of H and τ affects the convergence rate and accuracy of InvCM (Section 2.3).

### 2.2 Model Description

We use the atmospheric GCM ExoCAM, which is a modified version of the Community Atmospheric Model version 4 with a correlated‐k radiative transfer scheme that is accurate for high ${CO}_{2}$ mixing ratios and high temperatures. ExoCAM has been used for several climate studies, which demonstrate its capability of simulating extremely hot climates (Wolf, 2017; Wolf & Toon, 2013, 2014a, 2014b, 2015; Wolf et al., 2018, 2019, 2020). We use a resolution of 4°×5° horizontally and 40 layers vertically, with the model top extending to ∼1 mb. The atmosphere is coupled to a global immobile slab ocean, with zero assumed heat transport convergence. We do not include sea ice or continents in this study. The solar constant (1,361 W $m^{-2}$), the rotation period (23 h 56 min 4 s), and the length of the year (365 days) are fixed to present‐day Earth values. We set eccentricity and obliquity to zero, so there is no seasonal cycle in this study.

${CO}_{2}$ can affect the planetary climate in at least two ways. First, ${CO}_{2}$ induces radiative forcing on the planet. Second, at very high ${CO}_{2}$ mixing ratios, the thermodynamic properties of air are altered, affecting the temperature lapse rate and the heat transport efficiency of the atmosphere. Here, we only consider the radiative effect of ${CO}_{2}$. The reason is that modifying the total dry atmospheric mass or the molecular weight of dry air in the middle of a run is technically difficult in ExoCAM. We evolve the ${CO}_{2}$ mixing ratio in the following way. The thermodynamic properties of the dry air are all taken as those of $N_{2}$. The background surface $N_{2}$ pressure is 1 bar. Water vapor content is variable following the Clausius‐Clapeyron relation and water vapor availability. The total air pressure is the sum of $pN_{2}$ and $pH_{2}$O. The ${CO}_{2}$ mixing ratio, G, is accessible only to the radiative transfer module. The radiative transfer module considers the optical effects of $N_{2}$, $H_{2}$O, clouds, and ${CO}_{2}$. The density of ${CO}_{2}$ seen by the radiative transfer module is that of $N_{2}$ multiplied by G, which evolves following Equation 2. Note that G can be larger than 1 since it represents a mixing ratio.

We employ several groups of simulations with different mixed layer depths, equilibrium methods (InvCM or normal slab ocean simulations), ${CO}_{2}$ timescales (if InvCM is used), and initial states. All simulations mentioned in this article are tabulated in Table 1.

**Table 1:** List of all Simulations

Groups	Runs	Descriptions
*InvCM‐ClimateSensitivity*	51	The global mean SST is fixed to 290, 291, …, 340 K and the ${CO}_{2}$ mixing ratio evolves to equilibrate the system (see Section2 for details). To obtain the initial SST pattern and atmospheric conditions, we heat the slab ocean and the atmosphere of a modern climate uniformly by the difference between the fixed global‐mean temperature and that of the modern climate. The initial ${CO}_{2}$ mixing ratio is $18995\;\mu g\;g^{-1}$, which is the equilibrium ${CO}_{2}$ mixing ratio of a preliminary InvCM simulation under 310 K that is not listed in this table. Here, the modern climate means a north‐south symmetric and zonally uniform climate state, the SST pattern of which is lowest near the poles (−20.3°C), is highest on the equator (26.3°C), and has a global mean value of 11.9°C. The ${CO}_{2}$ timescale is 240 days and the mixed layer depth is 50 m. Each simulation runs for 15 yr. The equilibrium ${CO}_{2}$ as a function of the surface temperature is calculated via a polynomial regression of the geometric mean ${CO}_{2}$ of the last 10 yr with the random errors smoothed (See Section3.2 and Figure 6). Then we continue to run these simulations for 20 yr under the equilibrium ${CO}_{2}$ for the corresponding surface temperatures to examine whether equilibrium has been reached.
*NormalSlabOcean*	3	As in conventional slab‐ocean simulations: the ${CO}_{2}$ mixing ratio is fixed and the atmosphere is coupled to a slab ocean with a uniform depth of 50 m. We choose the ${CO}_{2}$ mixing ratios to be 18,407, 26,212, and 45,241 $\mu g\;g^{-1}$ based on the results from the *InvCM‐ClimateSensitivity* group so that the equilibrium global‐mean SST should be approximately 310, 320, and 330 K. The initial condition is a modern climate with a global surface temperature of 289 K. In order to be consistent with other simulations, the thermodynamic properties of the dry air are also taken as those of $N_{2}$ like in InvCM.
*InvCM‐Ensemble*	10	The same equilibrium technique is used as the *InvCM‐ClimateSensitivity* group. The global‐mean SST is set to 320 K for all simulations, and we modify the initial SST pattern for the 320 K simulation in the *InvCM‐ClimateSensitivity* group by adding $0.1\cos\theta \cos\left(\phi \;+\;\alpha_{i}\right)$ K, where θ and ϕ represent the latitude and the longitude and $\alpha_{i}$ is a random number between 0 and 2π, different for each case, so that this group constitutes an ensemble. We run each of the simulations for 10 yr.
*InvCM‐* ${CO}_{2}$ *Timescale*	4	The same equilibrium technique is used as the *InvCM‐ClimateSensitivity* group. The mixed layer depth is 50.0 m and the ${CO}_{2}$ timescale is 30, 60, 120, and 240 days. The global mean SST is set to 310 K for these 4 simulations. For the initial condition, we use the equilibrated 310 K simulation in the *InvCM‐ClimateSensitivity* group.
*InvCM‐MixedLayerDepth*	4	The same equilibrium technique is used as the *InvCM‐ClimateSensitivity* group. The ${CO}_{2}$ timescale is 240 days and the mixed layer depth is 0.5, 5, 50 and 100 m. The global mean SST is set to 310 K for these 4 simulations. We use the same initial condition as the 310 K simulation in the *InvCM‐ClimateSensitivity* group.
*InvCM‐InitCondSSTEquilibrium*	2	The same equilibrium technique is used as the *InvCM‐ClimateSensitivity* group. The ${CO}_{2}$ timescale is 240 days and the mixed layer depth is 50 m. The global mean SST is set to 310 K. One simulation (referred to as the control simulation) uses the same initial condition as the 310 K simulation in the *InvCM‐ClimateSensitivity* group. Another simulation (referred to as the experimental simulation) starts from the equilibrium SST pattern obtained from the control simulation and a ${CO}_{2}$ mixing ratio of $32768\;\mu g\;g^{-1}$, which is higher than the equilibrium value.
*InvCM‐FixedSSTPattern*	3	The SST is fixed everywhere. We allow the ${CO}_{2}$ mixing ratio to evolve as in the *InvCM‐ClimateSensitivity* group. The ${CO}_{2}$ timescale is 240 days and the mixed layer depth can be viewed as infinity. Three SST patterns are tested here. The first one is the equilibrium SST pattern in the *InvCM‐Ensemble* group, where the global‐mean SST is fixed to 320 K. Then we perturb this SST pattern with $\left(1\;K\right)\cdot Y_{2}^{0}$ and $\left(-1\;K\right)\cdot Y_{2}^{0}$ respectively to obtain the SST patterns with a lower or higher equator‐to‐pole SST difference.

### 2.3 Choice of Parameters for InvCM

We chose the parameters of InvCM, including the ${CO}_{2}$ timescale and the mixed layer depth, taking into account both the computational cost and the accuracy of InvCM. We perform two groups of simulations to find an appropriate set of parameters, one using a fixed mixed layer depth and varying the ${CO}_{2}$ timescale (the *InvCM‐* ${CO}_{2}$ *Timescale* group in Table 1) and the other using a fixed ${CO}_{2}$ timescale and varying the mixed layer depth (the *InvCM‐MixedLayerDepth* group in Table 1).

**Figure 1:**
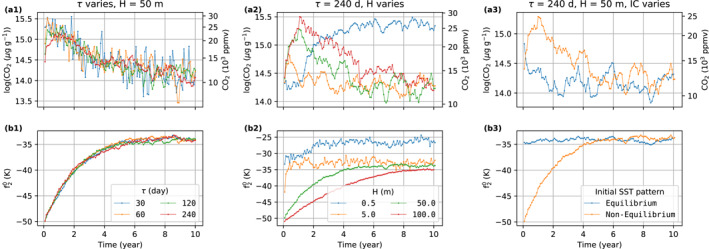
The choice of the ${CO}_{2}$ timescales does not affect the convergence rate of $f_{2}^{0}$, which is the speed bottleneck of InvCM. A small mixed layer depth can increase the convergence rate but may yield a different climate state. This figure shows the evolution of (a) ${CO}_{2}$ mixing ratio and (b) $f_{2}^{0}$, the expansion coefficient for $Y_{2}^{0}$ for (1) the *InvCM‐* ${CO}_{2}$ *Timescale* group, (2) the *InvCM‐MixedLayerDepth* group, and (3) the *InvCM‐InitCondSSTEquilibrium* group. The absolute value of $f_{2}^{0}$ reflects the meridional SST gradient. Note that we use the same initial condition for the *InvCM‐MixedLayerDepth* group. The initial $f_{2}^{0}$ looks different in Panel (b2) because we plot monthly averaged data here and a fast adjustment of $f_{2}^{0}$ happens due to a very low mixed layer depth.

We test ${CO}_{2}$ timescales of 30, 60, 120, and 240 days under the same mixed layer depth of 50 m. Figure 1a1 shows that the convergence rate of InvCM does not depend strongly on the ${CO}_{2}$ timescale. This is because the adjustment of the equator‐to‐pole SST difference limits the convergence speed. We describe the evolution of the SST pattern using a spherical harmonic expansion. We focus on the coefficients $f_{0}^{0}$, $f_{1}^{0}$, and $f_{2}^{0}$, which can be obtained in the following way:

(3) $$f_{l}^{0}=\int_{4\pi }T_{s}\left(\theta ,\phi \right)\cdot Y_{l}^{0}\left(\theta ,\phi \right)d\Omega$$

where

$$\begin{array}{ccc} Y_{0}^{0} & = & \frac{1}{2}\sqrt{\frac{1}{\pi }}\\ Y_{1}^{0} & = & \frac{1}{2}\sqrt{\frac{3}{\pi }}\sin\theta \\ Y_{2}^{0} & = & \frac{1}{4}\sqrt{\frac{5}{\pi }}\left(3\sin^{2}\theta -1\right) \end{array}$$

Here, θ represents the latitude.

$f_{0}^{0}$, $f_{1}^{0}$, and $f_{2}^{0}$ represent the global mean value, the north‐south asymmetry, and the equator‐to‐pole SST difference. Here, $f_{0}^{0}$ is fixed and there is no continent, sea ice or obliquity to force $f_{1}^{0}$ in our simulations, although $f_{1}^{0}$ can obtain a non‐zero value due to spontaneous symmetry breaking. The adjustment timescale of $f_{2}^{0}$ is a few years, independent of the ${CO}_{2}$ timescale (Figure 1b1). As a result, reducing the ${CO}_{2}$ timescale cannot speed up the convergence of the model if the ${CO}_{2}$ timescale is much shorter than the SST gradient response timescale. The ${CO}_{2}$ timescale can also affect the accuracy of InvCM. Figure 1a1 shows that the amplitude of the variation in ${CO}_{2}$ mixing ratio in the equilibrium state is greater when a smaller ${CO}_{2}$ timescale is used, which will result in greater random errors when evaluating the equilibrium ${CO}_{2}$ mixing ratio. We therefore use a ${CO}_{2}$ timescale of 240 days, which is the largest value we tested.

**Figure 2:**
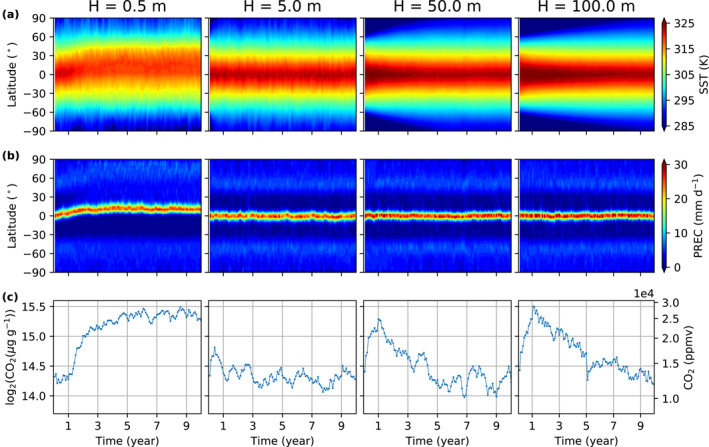
A thick slab ocean with high heat capacity stabilizes the ITCZ while a too small slab ocean depth, such as 0.5 m, results in a break of north‐south symmetry and thus affects the equilibrium ${CO}_{2}$ mixing ratio. See the evolution of (a) the zonal mean surface temperature, (b) precipitation, and (c) ${CO}_{2}$ mixing ratio for the *InvCM‐MixedLayerDepth* simulations. Here, the global mean SST is fixed to 310 K and the ${CO}_{2}$ timescale is 30 days.

The mixed layer depth can affect convection strength in local‐scale cloud‐resolving models and may modify the equilibrium climate state (Hohenegger & Stevens, 2016). We use a standard mixed layer depth of 50 m for two reasons. First, it is a reasonable approximation of the typically mixed layer depth in the ocean. Second, a mixed layer depth of 50 m results in a convergence timescale of 5 yr, which we think is fast enough. We examine how different mixed layer depths, including 0.5, 5, 50 and 100 m affect InvCM. The simulations with mixed layer depths of 5, 50, and 100 m take about 0.5, 5, and 10 yr to equilibrate. The fact that the convergence timescale is proportional to the mixed layer depth is consistent with the local SST adjustment being the convergence bottleneck. Interestingly, the simulation using a mixed layer depth of 0.5 m equilibrates at much higher ${CO}_{2}$ mixing ratio (Figure 1a2). This is associated with spontaneous symmetry breaking, with the highest temperature and precipitation rates occurring off the equator (Figure 2). While this could be an interesting topic of future research, in this article, we focus on simulations with 50 m mixed layer depth.

To confirm that local SST adjustment is the convergence bottleneck, we perform the *InvCM‐InitCondSSTEquilibrium* group of simulations (see Table 1 for the description). First, we run a control simulation using the same configuration as the 310 K simulation in the *InvCM‐ClimateSensitivity* group. For this simulation, the initial SST pattern is not in equilibrium but the initialized ${CO}_{2}$ mixing ratio ($18965\;\mu g\;g^{-1}$) is almost the same as the equilibrium value. Then, we perform an experimental simulation initialized with the equilibrium SST pattern of the control simulation in this group. This simulation equilibrates within 2 yr, which compares to 5 yr in the control simulation, even though we started it from a much higher ${CO}_{2}$ mixing ratio ($32768\;\mu g\;g^{-1}$). The equilibration timescale of 2 yr is about 3 times the ${CO}_{2}$ e‐folding timescale of 240 days, which is consistent with ${CO}_{2}$ convergence limiting convergence speed if the SST pattern is started in equilibrium. This simulation shows InvCM can be accelerated by making a guess at the equilibrium SST pattern, for example, by reducing the initial meridional SST gradient when simulating extremely hot climates.

## 3 Results

### 3.1 10x Speed‐Up and Accuracy of the InvCM Method

**Figure 3:**
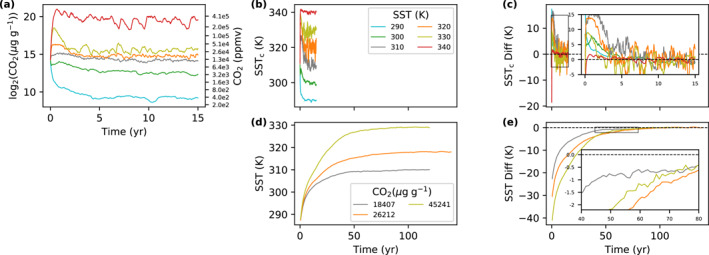
InvCM speeds convergence up by a factor of ten. This figure shows the time evolution of (a) ${CO}_{2}$ mixing ratio, (b) corresponding SST (${SST}_{c}$) for the real‐time ${CO}_{2}$ mixing ratio (see the first paragraph in Section 3.1 for the definition of corresponding SST), and (c) its difference with the fixed global mean SST in simulations with fixed global mean temperatures and prognostic ${CO}_{2}$ mixing ratios for several simulations in the *InvCM‐ClimateSensitivity* group. To compare the speed of convergence, the evolution of (d) SST and (e) its difference with the equilibrium SST of simulations in the *NormalSlabOcean* group are plotted on the same time axis. It takes ∼5 years for InvCM to equilibrate while the normal slab ocean simulations require 50–70 yr to equilibrate (defined as when the difference between the equilibrium and the current SST is less than 1 K).

Here, we show that InvCM is more than 10 times faster than normal slab ocean simulations. Figure 3a shows the evolution of ${CO}_{2}$ for a few members in the *InvCM‐ClimateSensitivity* group. See Table 1 for the configuration of this group of simulations. The convergence rates of InvCM and normal slab ocean simulations cannot be compared directly, because the two methods evolve different variables, ${CO}_{2}$ mixing ratio and global‐mean SST, to equilibrate the system. In order to make a comparison, we define the “corresponding SST” of a ${CO}_{2}$ mixing ratio, ${SST}_{c}$, as the global‐mean SST for which the equilibrium ${CO}_{2}$ is that value (Equation 4 and Figure 6a). That is to say we use our ensemble of *InvCM‐ClimateSensitivity* simulations to define a function that maps any equilibrium ${CO}_{2}$ to a “corresponding SST” in ExoCAM. In this way, the changes in ${CO}_{2}$ mixing ratio and the global‐mean SST can be linked through ECS for small enough changes. The difference between corresponding SST and the fixed global mean SST (shown in Figure 3c) converges to zero within ∼5 years. In contrast, normal slab ocean simulations for 310, 320, and 330 K take ∼45, ∼70, and ∼65 years, respectively, to equilibrate global‐mean SST to within 1 K.

A previous study using a zero‐dimensional climate model demonstrates that the e‐folding timescale for the surface temperature is proportional to the product of the heat capacity of the system and the temperature change per unit radiative forcing (Cronin & Emanuel, 2013). This timescale is much longer than the SST gradient response timescale because global radiative feedback is the only mechanism that equilibrates the global‐mean SST while both atmospheric heat transport and local radiative feedback contribute to adjusting the SST gradient. Since the SST gradient convergence rate is the bottleneck of InvCM (see our Section 2.3), the ratio between the convergence rates of the SST gradient and the global‐mean SST is the degree of speed‐up we gain from the InvCM method. The Cronin and Emanuel (2013) model also explain why the convergence timescale increases with the global‐mean SST. As the atmosphere warms, the water vapor content increases nearly exponentially, resulting in the buildup of latent heat in the atmosphere. For hot climates, the atmospheric heat capacity is comparable to, or even greater, than that of a slab ocean with a depth of 50 m. Additionally, the temperature change per unit radiative forcing is large for hot climates (Bloch‐Johnson et al., 2015; Bloch‐Johnson, Rugenstein, Stolpe, et al., 2020). The long equilibrium timescales we observe in our normal slab ocean simulations could be a result of a combination of the high heat capacity and the large temperature change per unit forcing. Notably, InvCM does not suffer from this phenomenon. In fact, InvCM converges even faster for extremely hot climates (cases of 330 and 340 K, see Figure 3c), which might be due to the fact that increased atmospheric latent heat transport leads to quicker evolution of the local SST.

**Figure 4:**
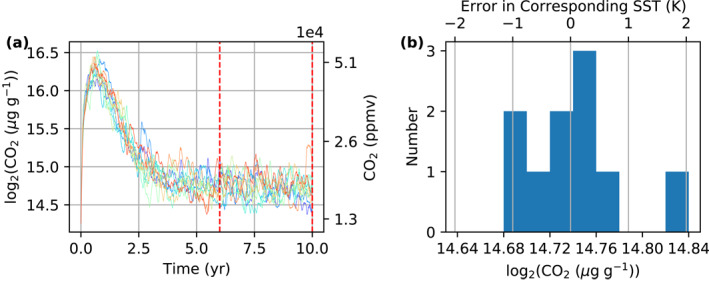
Our *InvCM‐Ensemble* group simulations show that when running the model for 10 yr with InvCM, the accuracy measured in the corresponding SST is about 1 K. This figure shows (a) the evolution of ${CO}_{2}$ mixing ratio for all 10 members in the *InvCM‐Ensemble* group described in Table 1 and (b) a histogram of the geometric mean of ${CO}_{2}$ mixing ratio in the last 4 yr (indicated by vertical red lines in Panel (a)) for each member. The deviation in the binary logarithm of ${CO}_{2}$ mixing ratio is mapped to that in the surface temperature with a scale factor of 20 K, which is the climate sensitivity at 320 K (see Section 3.2 and Figure 6 for climate sensitivity as a function of the global‐mean SST).

After equilibrium has been reached for InvCM, the ${CO}_{2}$ mixing ratio continues to fluctuate, and the amplitude of this fluctuation converted to the corresponding SST depends on the global‐mean SST and is largest for the cases of 310 and 320 K (Figures 3a and 3b). The largest amplitude is several Kelvin. This is larger than that of a normal slab ocean simulation in the same conditions and may result in considerable random errors for InvCM. We employ an ensemble of simulations to evaluate such random errors (see the *InvCM‐Ensemble* group in Table 1 for the configuration). Each simulation is run for 10 yr and we take the geometric mean ${CO}_{2}$ mixing ratio of the last 4 yr as the equilibrium value. Figure 4b shows that the error quantified as the corresponding SST is about 1 K. Only 1 out of 10 members in the ensemble has an error larger than 1 K. For the *InvCM‐Ensemble* group, the global‐mean SST is fixed to 320 K, where ECS is highest. A higher ECS is most likely to give bigger errors, so ±1K should be an upper bound of random errors for all SST values.

**Figure 5:**
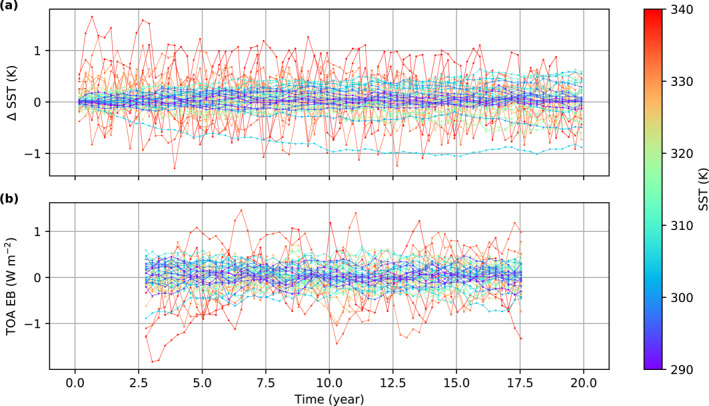
The equilibrium states produced by InvCM are very close to those of the normal slab‐ocean simulation. This figure shows (a) the deviation of SST from that InvCM uses and (b) the energy balance at TOA in branch runs from InvCM simulations using the normal slab‐ocean simulation method (see Table 1, the *InvCM‐ClimateSensitivity* group). We apply a 5 yr moving average to smooth the time‐series of the energy imbalance at TOA.

One way to determine the accuracy of InvCM is to compare the equilibrium climate states it produces to those produced by normal slab‐ocean simulations. For each simulation in the *InvCM‐ClimateSensitivity* group, we branch a normal slab‐ocean run from it. For all branch runs, the deviation of SST is smaller than 1 K (Figure 5). Some extremely hot simulations exhibit strong variation in SST but show no trend in global‐mean SST. How close a state is to equilibrium can also be measured by the TOA energy balance. All branch runs have variations in TOA energy balance less than 1 W $m^{-2}$. This also demonstrates an alternative use of InvCM: it could be used to find states roughly in equilibrium quickly before switching back to a normal slab‐ocean simulation. In this way, it would be possible to benefit from the speedup of InvCM and not have to worry about possible systematic bias or a potentially unrealistic configuration.

### 3.2 ECS Bump at High‐Temperature Resolution

We investigate the evolution of the climate from 290 to 340 K at an interval of 1 K using 51 simulations (see Table 1, the *InvCM‐ClimateSensitivity* group). First, we focus on the relationship between the equilibrium ${CO}_{2}$ and SST. Then, we look at the evolution of the distributions of SST, OLR, and ASR.

**Figure 6:**
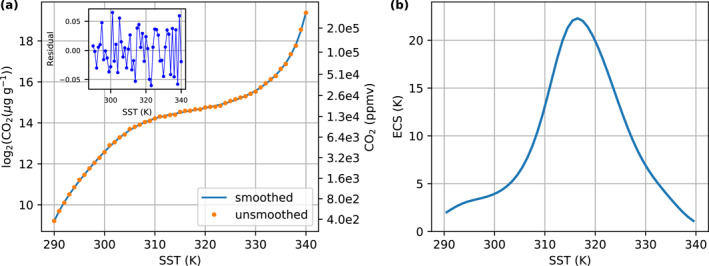
Climate sensitivity maximizes near 315 K and the maximum climate sensitivity is above 20 K per doubling ${CO}_{2}$. This figure shows (a) the equilibrium ${CO}_{2}$ mixing ratio for different global mean SSTs. By fitting a 10° polynomial to the geometric mean of ${CO}_{2}$ mixing ratio of the last 10 yr for each global mean SST depicted by orange points, we obtain the smoothed relation between the equilibrium ${CO}_{2}$ as a function of SST (the blue line). (b) Climate sensitivity calculated with the smoothed data following Equation 5.

The equilibrium ${CO}_{2}$ can be obtained as a function of the global mean SST (see Figure 6a). We use a 10° polynomial regression to smooth the ${CO}_{2}$ as a function of surface temperature in the following form:

(4) $$G_{Eq}\left(T\right)=\sum_{n=0}^{10}a_{n}T^{n}$$

All of the data points lie close to the polynomial fit (Figure 6a). There is no trend or obvious structure in the residuals and the absolute values of the residuals (no more than ∼0.05 doublings of ${CO}_{2}$) are small compared to the overall change in the ${CO}_{2}$ mixing ratio among the *InvCM‐ClimateSensitivity* group. Moreover, the amplitude of the residuals is of similar magnitude to the random errors in our *InvCM‐Ensemble* simulations (Figure 4b), so these residuals can be accounted for as random errors due to natural variation. We define an infinitesimal climate sensitivity in the following way:

(5) $$ECS\left(T\right)=\frac{d\;T}{d\log_{2}G_{Eq}}$$

where $G_{eq}$ is the equilibrium ${CO}_{2}$ mixing ratio as a function of the global‐mean SST obtained through a polynomial fit mentioned above. The advantage of this definition is that it defines ECS as a local derivative as opposed to a finite difference, but it still reduces to the usual definition when ECS is constant. In particular:

$$\begin{array}{ccc} T(2G_{0})-T(G_{0}) & = & \int_{G_{0}}^{2G_{0}}d\;T\\ & = & \int_{G_{0}}^{2G_{0}}\mathrm{ECS}\;d\log_{2}G_{\text{Eq}}\\ & = & \mathrm{ECS} \end{array}$$

Figure 6b depicts climate sensitivity as a function of SST. The climate sensitivity maximizes near 315 K and the maximum is more than 20 K per doubling of ${CO}_{2}$.

**Figure 7:**
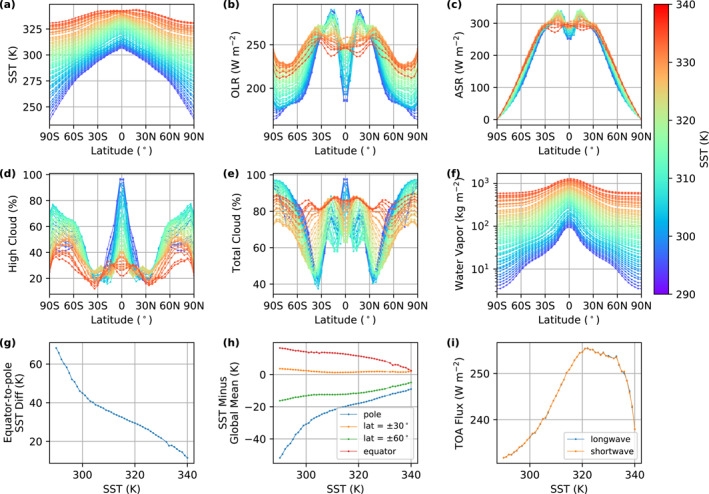
The meridional SST decreases dramatically when the climate warms. This figure shows the equilibrium zonal mean (a) SST, (b) OLR, (c) ASR, vertically integrated (d) low and (e) high cloud fraction, (f) vertically integrated water vapor, (g) equator‐to‐pole SST difference, (h) SST at the several latitudes, and (i) global‐mean OLR and ASR as a function of the global‐mean SST.

The spatial response to the increase of the global mean SST can be captured by InvCM. When the climate warms from 290 to 340 K, the equator‐to‐pole SST difference decreases significantly (See Figures 7a and 7g). For extremely hot climates (340 K), the equator‐to‐pole temperature difference is as low as ∼10K. Additionally, the SST between 30°S and 30°N is nearly uniform in this case (Figure 7h). Interestingly, the SST pattern exhibits a small amount of north‐south asymmetry for extremely hot climates (Figure 7a). The SST at a latitude of 30° is always close to the global‐mean SST, being only a few degrees higher for a wide range of global mean SST of 50 K. This may not be a coincidence, since half of the area of the planet is equatorward of 30° and half is poleward. Note that the SST can be lower than the freezing point of sea water because we have turned off sea ice for all simulations here.

The evolution of the OLR and ASR patterns is more complicated. The OLR at the equator increases dramatically until the global‐mean SST reaches ∼325 K while the subtropical OLR (between 15° and 30°) decreases (Figures 7b and 7c). This trend can be explained by a weakening Hadley circulation. Weaker upwelling at the equator reduces the cloud coverage there and thus results in both higher OLR and ASR. Meanwhile, weaker descent in the subtropics and a warming climate leads to more water vapor and reduces the OLR there (Figures 7d–7f). The positive radiative‐convective feedback that Wolf and Toon (2015) described might be the cause of the dramatic decrease in high cloud coverage as SST goes from 310 to 330 K. The global‐mean OLR and ASR as a function of SST show a sawtoothed shape with a maximum around ∼320 K (see Figure 7i). This is similar to the planetary albedo trend reported by Wolf and Toon (2015). There is likely also a clear‐sky contribution, as argued by Seeley and Jeevanjee (2021).

### 3.3 Hysteresis and the Onset Runaway Greenhouse

Here, we use a zero‐dimensional energy balance model (EBM) to demonstrate the way InvCM could be applied in future GCM studies to fully explore a global climate hysteresis diagram and to investigate climate states very near to the transition to a runaway greenhouse. In our first iteration of the EBM, we define the TOA net downwelling energy flux, $N\left(T_{s},G\right)$, as:

(6) $$N\left(T_{s},G\right)=\lambda \left(T_{s}-T_{0}\right)+A_{0}\log_{2}\left(G/G_{0}\right)-\frac{1}{4}\left(\alpha_{p}\left(T_{s}\right)-\alpha_{p}\left(T_{0}\right)\right)S_{0}$$

where $T_{s}$ is the surface temperature, $T_{0}$ is the reference surface temperature (290 K), λ is the climate feedback (−1.17 W $m^{-2}$ $K^{-1}$), $A_{0}$ is the climate forcing parameter ($3.7\;\;W\;m^{-2}$), G is the greenhouse gas mixing ratio, $G_{0}$ is the reference greenhouse gas mixing ratio, $\alpha_{p}$ is the planetary albedo, and $S_{0}$ is the solar radiative flux (1,361 W $m^{-2}$). We evolve either $T_{s}$ or G in order to force $N\left(T_{s},G\right)$ to zero. When we evolve $T_{s}$, the EBM is analogous to the normal method of converging a GCM. When we evolve G, the EBM is analogous to the InvCM method of converging a GCM.

First, we consider a climate that can contain hysteresis. The sea‐ice feedback has been known to result in hysteresis in planetary climate for a long time (Abbot et al., 2018; Budyko, 1969; Sellers, 1969; Yang et al., 2017), while several recent studies found hysteresis in warm climates due to a positive cloud feedback (Popp et al., 2016; Schneider et al., 2019). Here, we assume that the planetary albedo is specified by the following equation, possibly as a result of changes in stratiform clouds (Schneider et al., 2019):

(7) $$\alpha_{p}\left(T_{s}\right)=0.3-0.05\tanh\left(\frac{T_{s}-310K}{T_{w}}\right)$$

**Figure 8:**
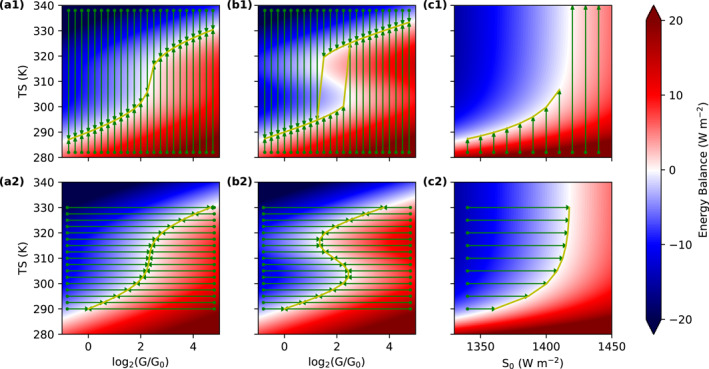
InvCMenables us to find more climate states, even those which are unstable or undergoing the runaway greenhouse effect. These subplots show energy balance as a function of the surface temperature ($T_{s}$) and an influencing factor of the climate (G for ${CO}_{2}$ and $S_{0}$ for the solar constant). The arrows in green indicate the evolution of the state. The first row is for normal slab ocean simulations, where $T_{s}$ evolves, and the second row is for InvCM, which requires ${CO}_{2}$ mixing ratio (or $S_{0}$) to adjust. The yellow lines represent the equilibrium relationship between $T_{s}$ and ${CO}_{2}$ mixing ratio (or $S_{0}$).

$T_{w}$ is the transition temperature scale for the assumed change in stratiform clouds. The smaller $T_{w}$ is, the more rapid the cloud transition is. If we set $T_{w}$ = 15 K, there is no climate hysteresis and the equilibrium climate state does not depend on the variable, we choose to evolve (Figure 8a). When we reduce $T_{w}$ to 10 K, in contrast, climate hysteresis results (Figure 8b). There is now a range of the forcing parameter where three climate states are possible: two stable and one unstable. Critically, we can equilibrate the EBM for all three climate states when we evolve G (InvCM, Figure 8b2), but we can only equilibrate the EBM for the stable climate states when we evolve $T_{s}$ (normal method, Figure 8b1). This motivates us to speculate that InvCM could be used to fully specify a climate hysteresis diagram for a GCM by finding unstable climate states.

Next, we study a climate near the transition to a runaway greenhouse state. The runaway greenhouse is a result of the saturation of the negative longwave feedback at high temperatures due to the strong water‐vapor feedback (Ingersoll, 2013). We modify our specification of the TOA energy imbalance in our EBM to represent this behavior as follows:

(8) $$N\left(T_{s},S\right)=F_{m}\left(\exp\left(-|\lambda |\frac{T_{s}-T_{0}}{F_{m}}\right)-1\right)+\frac{1}{4}\left(1-\alpha_{p}\right)\left(S-S_{0}\right)$$

where $F_{m}=10\;W\;m^{-2}$, $|\lambda |=1.17\;W\;m^{-2}\;K^{-1}$, $\alpha_{p}=0.3$, $S_{0}=1361\;W\;m^{-2}$.

Because the transition to a runaway greenhouse state occurs over a narrow range in S, it is very difficult to find climate states near this transition by evolving $T_{s}$ (Figure 8c1). In contrast, when we fix $T_{s}$ and evolve S (InvCM), we can equilibrate the climate to any $T_{s}$ we desire (Figure 8c2). This allows us to sample climate states that are very near to the runaway greenhouse state, but would be extremely difficult to find by running the model using the normal method. We speculate that InvCM could therefore be useful for finding climate states near the transition to a runaway greenhouse in a GCM, which would allow us to better understand the circulation, cloud, and moisture processes that result in this regime. Furthermore, this technique could be applied near any other climate transition where the climate sensitivity becomes large over a small range of the forcing parameter.

## 4 Discussion

### 4.1 Equilibrating the Model for a Given SST Pattern

**Figure 9:**
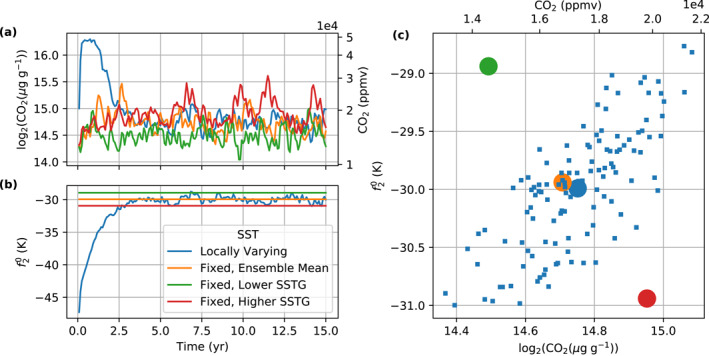
The *InvCM‐ClimateSensitivity* simulations show a strong correlation between the meridional SST gradient (proportional to the absolute value of $f_{2}^{0}$) and the ${CO}_{2}$ mixing ratio in the *InvCM‐ClimateSensitivity* group (here, we only focus on the case with its global‐mean SST fixed to 320 K), but the direction is different from that in fixed‐SST simulations. The fixed‐SST simulations are described in Table 1. See the evolution of (a) the ${CO}_{2}$ mixing ratio and (b) the SST expansion coefficient for $Y_{2}^{0}$. The circles in Panel (c) present the mean of the logarithm of ${CO}_{2}$ mixing ratio and $f_{2}^{0}$ of the last 10 yr in each simulation while the squares present monthly data in the *InvCM‐ClimateSensitivity* simulation of the same period of time.

For hot simulations in the *InvCM‐ClimateSensitivity* group, the time‐series of both ${CO}_{2}$ mixing ratio and the meridional SST gradient are noisy. We observe a strong correlation between these two variables in any of these simulations (the 320 K case is depicted in Figure 9, blue lines in (a) and (b) and squares in (c), *r* = 0.75). In order to determine whether there is a causal relationship between these variables, we performed a set of simulations (*InvCM‐FixedSSTPattern* group) with fixed SST patterns to see if prescribing the SST pattern affects the equilibrium ${CO}_{2}$ mixing ratio. We have 3 simulations in this group with varying equator‐to‐pole SST gradients (See Table 1 for details).

For the *InvCM‐FixedSSTPattern* simulations, the amplitude of variation of ${CO}_{2}$ in the equilibrated state is slightly larger than for the *InvCM‐ClimateSensitivity* group (Figure 9a). This suggests that the variation in ${CO}_{2}$ mixing ratio in the *InvCM‐ClimateSensitivity* simulations is not caused primarily by fluctuation in the meridional SST gradient. More interestingly, when we perturb the fixed‐SST pattern with a higher or lower SST gradient, the equilibrium ${CO}_{2}$ changes, but in the opposite direction to that observed in the *InvCM‐ClimateSensitivity* simulations. Specifically, simulations with a higher imposed SST gradient have a higher equilibrium ${CO}_{2}$ mixing ratio (Figure 9). It is therefore clear that this is not the only causal relationship at work, and additional feedbacks and processes must be operative. One possible explanation is that the correlation is caused by a third factor. This third factor could be the tropical low cloud cover, which increases the ${CO}_{2}$ mixing ratio by reflecting more shortwave radiation and decreases the meridional SST gradient by cooling the tropical ocean. Future studies might provide a comprehensive understanding of the source of the natural fluctuations in these variables and the interactions between them.

This group of fixed‐SST simulations also demonstrates the capability of using the InvCM method to equilibrate ${CO}_{2}$ for a specified SST pattern. Here, we focus on the meridional SST gradient because we use an aquaplanet configuration in which any zonal asymmetry will be temporary. For the Earth's continental configuration, atmospheric overturning circulations due to strongly zonally asymmetric features are interesting and important topics. Several recent studies investigated the effect of spatial patterns of warming on the TOA radiative fluxes (Bloch‐Johnson, Rugenstein, & Abbot, 2020; Dong et al., 2019; Zhou et al., 2017). InvCM using a fixed SST pattern could be useful in future studies of the spatial effect of radiative feedbacks. We could even see how the function $ECS\left(p{CO}_{2}\right)$ (or ECS(T)) in turn depends on continental configuration by running a number of such experiments, the number of which could be large as a result of the speed‐up that InvCM provides.

### 4.2 Link to Conventional Climate Modeling

InvCM is an inverse method. Although it does not reveal how the climate responds to forcing directly, it resolves the feedback of the atmosphere due to the change in the global‐mean SST and thereby can be used to indirectly find the value of the corresponding forcing. Additionally, InvCM can more easily capture climate states near bifurcations or instability, as pointed out in Section 3.3.

The specific results of normal GCM simulations are not necessarily ends in themselves, in that we do not expect that humanity will exactly quadruple the ${CO}_{2}$ concentration relative to the preindustrial, or exactly follow any of the emissions scenarios explored in CMIP. Instead, these simulations help us broadly understand the relationship between forcing and response, providing specific instances of a more general mapping. Inverse simulations do the same thing, while running more quickly. In this sense, inverse simulations are no more sensitive to the SST chosen than normal simulations are to ${CO}_{2}$ concentration chosen—they will both provide similar information about the connection between forcing and response, and allow for similar interpolations to nearby values.

Here, we have implemented InvCM in a relatively simple model configuration (no seasonal cycles and a slab ocean) that is advantageous for theoretical planetary climate studies with GCMs, but unsuitable for realistic near‐future climate forecasts. When studying planetary climate, simple climate configurations are often used (Kaspi & Showman, 2015; Komacek & Abbot, 2019; Wolf, 2017; Wolf et al., 2018, 2020; Yang et al., 2013). Moreover, studies sometimes use slab oceans in GCM simulations to investigate the impact of global warming, focusing particularly on the role of the atmosphere (Lu & Cai, 2010; Park et al., 2018). The InvCM method can accelerate similar simulations using a slab ocean. More importantly, the cost of near‐global cloud‐resolving simulations using a slab‐ocean with interactive SST could be drastically decreased using the InvCM method. This is important because although increasingly powerful supercomputers have made near‐global cloud‐resolving simulations possible, most such simulations currently still use a prescribed SST pattern (Bretherton & Khairoutdinov, 2015; Narenpitak et al., 2017, 2020; Tomita et al., 2005).

### 4.3 Possible Extensions and Future Work

In our simulations, the atmosphere is coupled to a global slab ocean with a uniform depth. However, in principle the method we have developed could potentially be modified to incorporate land or sea ice, or to extend the slab ocean to one with spatially varying depths. In this case, it might be advantageous to fix the energy of the ocean instead of the SST. If this is done, the global mean surface temperature may not be constant.

InvCM might also be adapted for a dynamic ocean by applying multiple fluxes at multiple levels. This would address the large heat capacity of a deep ocean, and could lead to a significant decrease in model convergence time. However, the thermohaline circulation is so slow that it can still take thousands of years for a water parcel to close the global overturning circulation, which could limit the decrease in model convergence time. Future research is necessary to determine which effect is more important.

We do not consider seasonal cycles in our simulations. If either the eccentricity or obliquity is nonzero, the seasonal distribution of the shortwave flux will vary. In this case, for normal simulations, the global mean SST may not converge to a constant value, but might instead oscillate in response to the periodic external forcing after it reaches equilibrium. Since InvCM constrains the global mean SST to be constant, it will not typically create the same climate under seasonally varying insolation as a normal simulation. Moreover, since we evolve the ${CO}_{2}$ mixing ratio to adjust the TOA energy flux to zero, the ${CO}_{2}$ mixing ratio may oscillate periodically. To reduce the amplitude of this oscillation, we would need to make ${CO}_{2}$ time scale long compared to a seasonal cycle. As a result, it is possible that InvCM would lose its speed‐up advantage, although it would still enable us explore unstable states of an equilibrium curve. Alternatively, if the seasonal forcing is relatively small, we can first run the InvCM simulation until it equilibrates and then uses this state as the initial condition for a normal simulation. Future research is needed to determine the effectiveness of either approach.

## 5 Conclusions

We have applied the InvCM equilibration method to the GCM ExoCAM run with a mixed layer ocean. InvCM fixes the global‐mean SST and evolves the ${CO}_{2}$ mixing ratio and SST pattern until an equilibrium is reached. Our main conclusions are:

1. InvCM produces the same climate in ExoCAM as normal slab‐ocean simulations with fixed ${CO}_{2}$
2. InvCM converges about 10 times faster than normal slab‐ocean simulations
3. The speed of InvCM allows us to investigate the climate sensitivity for doubling ${CO}_{2}$ for global mean surface temperatures ranging from 290 to 340 K at an interval of 1 K. We reproduce the climate sensitivity bump near 315 K at a much higher temperature resolution. This will allow more detailed study of the cause of the climate sensitivity bump
4. InvCM has the potential to find unstable climate states and climate states that are close to the transition to a runaway greenhouse

## Data Availability

The model output used in this study and our modification to the original ExoCAM model are publicly available at the Knowledge@Uchicago internet database (http://dx.doi.org/10.6082/uchicago.3417). ExoCAM, the numerical model the authors used in this study, is available on GitHub at https://github.com/storyofthewolf/ExoCAM and https://github.com/storyofthewolf/ExoRT. The authors have also uploaded our modification to the original ExoCAM model on GitHub at https://github.com/Yixiao-Zhang/evolving-CO2-in-ExoCAM.
